# FN1 and TGFBI are key biomarkers of macrophage immune injury in diabetic kidney disease

**DOI:** 10.1097/MD.0000000000035794

**Published:** 2023-11-10

**Authors:** Fulin Dou, Qingzhen Liu, Shasha Lv, Qiaoying Xu, Xueling Wang, Shanshan Liu, Gang Liu

**Affiliations:** a Department of Nephrology, The Second Hospital of Shandong University, Jinan, China; b Nephrology Research Institute of Shandong University, The Second Hospital of Shandong University, Jinan, China.

**Keywords:** bioinformatics, diabetic kidney disease, immune injury, machine learning algorithm, macrophages, WGCNA

## Abstract

The pathogenesis of diabetic kidney disease (DKD) is complex, and the existing treatment methods cannot control disease progression well. Macrophages play an important role in the development of DKD. This study aimed to search for biomarkers involved in immune injury induced by macrophages in DKD. The GSE96804 dataset was downloaded and analyzed by the CIBERSORT algorithm to understand the differential infiltration of macrophages between DKD and normal controls. Weighted gene co-expression network analysis was used to explore the correlation between gene expression modules and macrophages in renal tissue of DKD patients. Protein-protein interaction network and machine learning algorithm were used to screen the hub genes in the key modules. Subsequently, the GSE30528 dataset was used to further validate the expression of hub genes and analyze the diagnostic effect by the receiver operating characteristic curve. The clinical data were applied to explore the prognostic significance of hub genes. CIBERSORT analysis showed that macrophages increased significantly in DKD renal tissue samples. A total of ten modules were generated by weighted gene co-expression network analysis, of which the blue module was closely associated with macrophages. The blue module mainly played an important role in biological processes such as immune response and fibrosis. Fibronectin 1 (FN1) and transforming growth factor beta induced (TGFBI) were identified as hub genes of DKD patients. Receiver operating characteristic curve analysis was performed in the test cohort: FN1 and TGFBI had larger area under the curve values (0.99 and 0.88, respectively). Clinical validation showed that 2 hub genes were negatively correlated with the estimated glomerular filtration rate in DKD patients. In addition, FN1 and TGFBI showed a strong positive correlation with macrophage alternative activation. FN1 and TGFBI are promising biomarkers for the diagnosis and treatment of DKD patients, which may participate in immune response and fibrosis induced by macrophages.

## 1. Introduction

Diabetic kidney disease (DKD) is the major cause of end-stage renal disease,^[1]^ which brings a heavy economic burden to patients and society. At present, there is no specific therapy for DKD. The existing treatment strategies aim to control blood glucose and blood pressure levels and inhibit the renin-angiotensin system. However, it has been proved that this approach can only delay the disease progression, but cannot prevent or reverse the disease.^[2]^ Furthermore, due to the genetic heterogeneity and complexity of the disease, not all patients benefit from these drugs.^[3]^ Therefore, it is urgent to improve the understanding of the pathogenesis of DKD to identify new potential diagnostic and therapeutic targets.

The immune response is involved in the pathogenesis and progress of DKD.^[4]^ In renal biopsy specimens of DKD patients at various stages, the infiltration of immune cells and increased expression of inflammatory mediators have been observed, among which macrophages are the most important immune cells.^[5]^ The accumulation of macrophages in the kidney is closely related to urinary albumin excretion, estimated glomerular filtration rate (eGFR), and interstitial fibrosis score. A body of evidence supports that^[6,7]^ macrophages induce renal injury through interacting with resident renal cells or being activated by components of the diabetic environment, resulting in the production of a large number of proinflammatory and profibrogenic factors. In addition, due to the plasticity of macrophages, they could acquire different phenotypes, namely classical activation (M1) or alternative activation (M2). The function of M1 is to promote immune inflammation leading to tissue damage, while M2 is primarily involved in tissue healing and inflammation regression, but some M2 may become to promote fibrosis.^[8]^ Little is known about how M2 terminates the repair response and starts fibrosis. Depletion of macrophages significantly reduces proteinuria and glomerulopathy in diabetic mice.^[9]^ Although many preclinical studies have shown that^[10,11]^ targeted therapy of the innate immune pathway in DKD may have a renoprotective effect, nonspecific anti-inflammatory therapy for DKD may increase the susceptibility to infection and it is not suitable for the DKD population with immune dysfunction.^[12]^ Therefore, we should actively explore biomarkers related to macrophage infiltration activation and phenotypic regulation of DKD to provide targets for more specific and less toxic treatment in the future.

**Figure 1.**
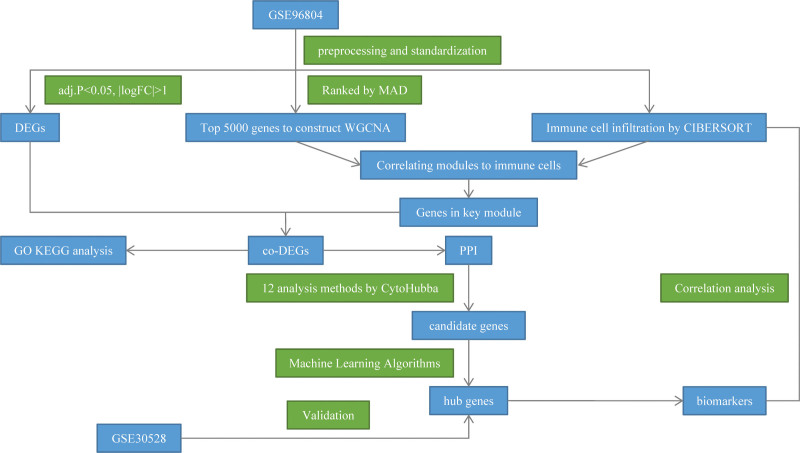
Study flowchart (DEGs = differentially expressed genes. MAD = median absolute deviation; WGCNA = weighted gene co-expression network analysis; GO = Gene Ontology; KEGG = Kyoto Encyclopedia of Genes and Genomes; PPI = protein-protein interaction).

To explore the key biomarkers involved in the immune damage of DKD caused by macrophages, our study first applied the CIBERSORT algorithm to confirm that macrophage infiltration in DKD samples increased significantly. Afterward, the CIBERSORT algorithm was combined with weighted gene co-expression network analysis (WGCNA) to find the gene co-expression modules highly related to macrophages. Protein-protein interaction (PPI) analysis and machine learning algorithm were performed on the genes in the module to identify the 2 hub genes in DKD. Finally, the GSE30528 dataset from the gene expression omnibus (GEO) database was used to validate the good diagnostic efficacy and prognostic value of hub genes (Fig. 1).

In this study, we aimed to identify potential biomarkers that modulate macrophages’ exacerbation of immune damage in DKD by integrating immune infiltration analysis, WGCNA and machine learning algorithms, providing a new viewpoint for the diagnosis and treatment of DKD.

## 2. Materials and methods

### 2.1. Data source and analysis

GSE96804^[13]^ and GSE30528^[14]^ were obtained from the GEO database. GSE96804 and GSE30528 were annotated based on the GPL17586 and GPL571 platform files, respectively. Among them, GSE96804 was used as the training dataset, including 41 DKD samples and 20 normal samples, and GSE30528 was used as the validation, including 9 DKD samples and 13 normal samples. All these samples were from human glomerular tissue and were expression profiling by array. The “arrayqualitymetrics” package^[15]^ in R was used to evaluate the training dataset and remove outliers, followed by the “oligo” package^[16]^ to standardize the dataset. “Limma” package^[17]^ in R was used to screen differentially expressed genes (DEGs) between DKD and normal samples with adj. *P* < .05, |Log2 fold change|>1. The results were visualized as volcano plot and heatmap by “ggplot2”,^[18]^ “pheatmap” package.^[19]^

### 2.2. Immune cell infiltration analysis

CIBERSORT method^[20]^ in R was used to further analyze the gene expression matrix of GSE96804, and to explore the differences in the composition of 22 immune cells between DKD and normal kidney tissue samples. The results were visualized by the “ggplot2,”^[18]^ “ggpubr” and “pheatmap” packages^[19]^ in R.

### 2.3. Construction of gene co-expression network module and identification of key modules

To screen the potential genes associated with DKD, we first ranked the genes by the median absolute deviation and included the top 5000 genes. Afterward, the WGCNA package^[21]^ in R was applied to construct the co-expression network modules by merging modules with the degree of dissimilarity <0.25 and setting the minimum number of genes in the module to 30. Then, we explored the correlation between gene expression modules and DKD, monocytes/macrophages. Ultimately, a key module was confirmed based on the *P* value and the correlation coefficients in macrophase. The overlapping genes of key module and DEGs were selected as target genes for subsequent analysis.

### 2.4. Enrichment analysis of the co-DEGs

In this study, DAVID (https://david.ncifcrf.gov/)^[22]^ tool was used for gene ontology (GO) functional enrichment analysis and Kyoto encyclopedia of genes and genomes (KEGG) pathway analysis for co-DEGs. The number of enrichment genes (count number) ≥4 and *P* value < .05 were chosen as cutoff criteria.

### 2.5. Construction of protein interaction networks and identification of candidate genes

The STRING software^[23]^ (version 10.0; https://string-db.org/) was applied to construct the PPI networks of co-DEGs, where the comprehensive score threshold was >0.4. Cytoscape software^[24]^ was used to visualize the above results and the plug-in cytohubba was applied to explore important nodes in biological networks. We ranked all nodes by the 12 topological analysis methods provided by CytoHubba. Each algorithm computed all node scores, and then 1 to 50 points were assigned based on the rank. According to all points, we selected the top 20 genes as candidate genes.

### 2.6. Identification of hub genes for DKD based on Machine Learning Algorithms

First, we used the least absolute shrinkage and selection operator (LASSO) logistic regression algorithm^[25]^ by the “glmnet” R package^[26]^ to screen these candidates for potential genes. Then, we applied the support vector machines (SVM)-recursive feature elimination (RFE) algorithm^[27]^ and the random forest (RF) algorithm to filter these candidates again based on the “e1071” R package^[28]^ and the “randomforest” R package,^[29]^ respectively. Finally, overlapping genes among potential genes generated via LASSO, SVM-RFE and RF algorithms were considered hub genes in the DKD.

### 2.7. Diagnostic efficacy and prognostic risk assessment of hub genes

First, we downloaded the GSE30528 dataset from the GEO database for cross-validation, compared the log2 transformation mRNA expression level of hub genes between the DKD and the control group, and used receiver operator characteristics (ROC) analysis to evaluate the discrimination ability of hub genes to DKD by calculating the area under the curve (AUC). The AUC value was calculated by the “pROC” package.^[30]^ After that, the clinical data of the validation dataset GSE30528 was obtained from the “Nephroseq” online platform (http://v5.nephroseq.org) to explore the Pearson correlation between the mRNA expression levels of hub genes and eGFR values. Finally, we analyzed the correlation between hub genes and 22 immune cell types.

## 3. Results

### 3.1. Identification of DEGs

**Figure 2.**
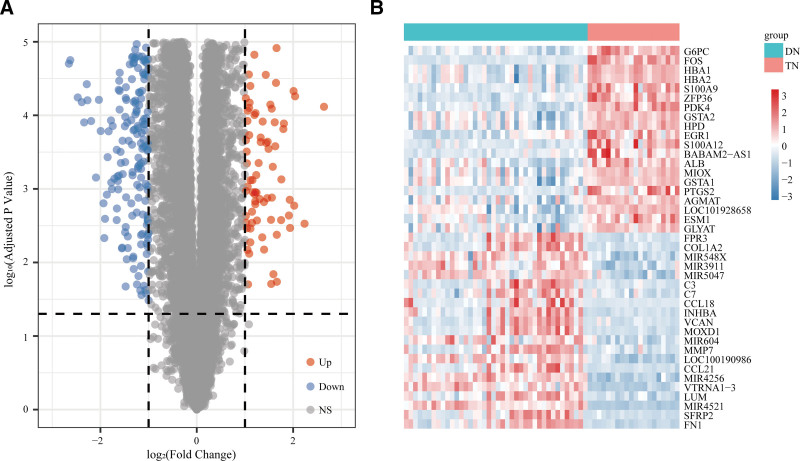
Identification of differentially expressed genes (DEGs) in the GSE96804 dataset. (A) Volcano plot and (B) heatmap of DEGs in GSE96804.

The outlier sample GSM2544307 was deleted from the GSE96804 dataset and finally, 40 cases of DKD and 20 cases of normal were included. A volcano plot was used to show 490 DEGs screened by GSE96804, of which 176 up-regulated genes and 314 down-regulated genes. We used heatmap to show the DEGs of the top 20 up and down (Fig. 2A and B).

### 3.2. Immune cell infiltration characteristics

**Figure 3.**
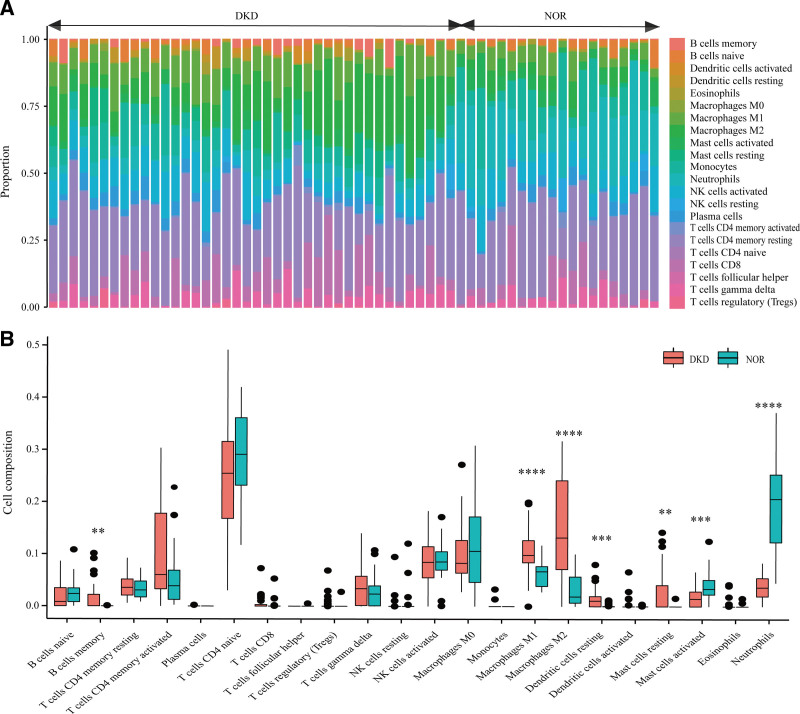
Immune cell infiltration differs between DKD and normal tissues. (A) Proportions of immune cell subsets in DKD and normal groups. B) Statistical differences in immune cell subsets in DKD and normal groups. * *P* < .05, ** *P* < .01, *** *P* < .001, **** *P* < .0001. DKD = diabetic kidney disease.

Our results more intuitively showed the proportion of various immune cell compositions in each sample (Fig. 3A), as well as the immune cell infiltration with a significant difference between the 2 groups (*P* < .05). M1, M2, memory B cells, and resting mast cells increased in the DKD group, while activating mast cells and neutrophils increased in the normal group (Fig. 3B).

### 3.3. Co-expression network and key modules

**Figure 4.**
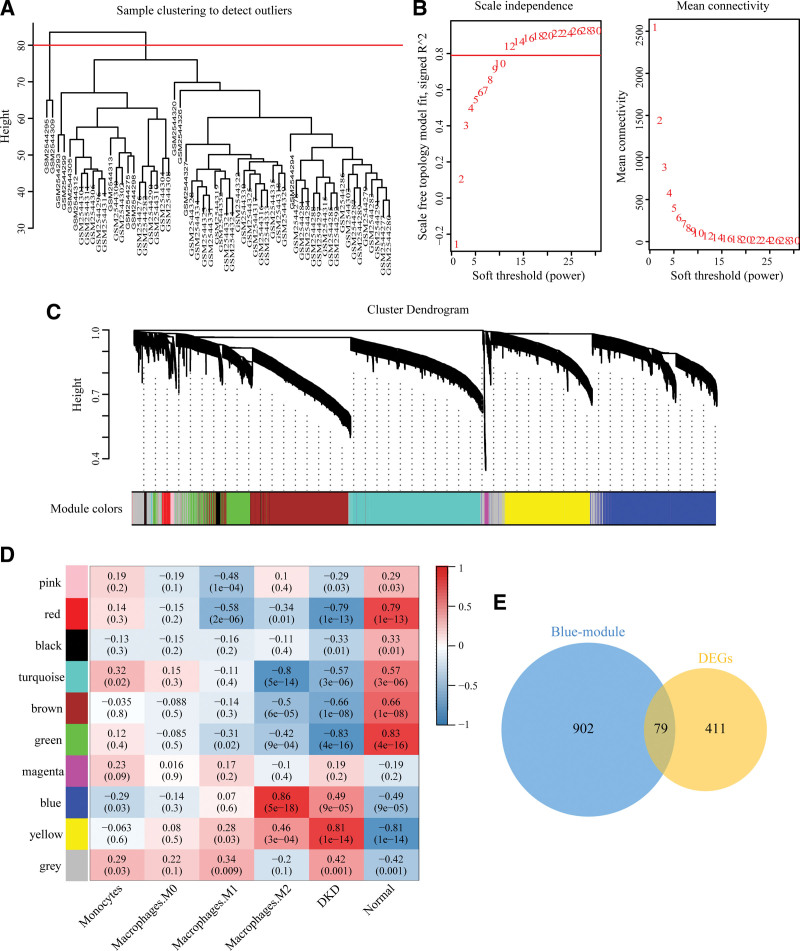
Construction of the WGCNA and identification of key module. (A) Sample clustering diagram (set height to 80 and remove 2 outliers). (B) Determine the optimal soft threshold powers according to the scale free topology model fit (R2) and mean connectivity, β = 12. (C) Clustering tree of coexpressive gene modules. Each branch represents 1 gene, and each color below represents 1 co-expression module. (D) Correlation between modules and DKD, monocytes/macrophages. Correlation coefficient and *P* value are marked in each grid. (E) Venn diagram shows the overlapping genes of differentially expressed genes (DEGs) and blue module, co-DEGs. WGCNA = weighted gene co-expression network analysis.

The clustering analysis of all samples showed that GSM2544295 and GSM2544309 samples were poorly clustered, so the above outlier samples were removed in the subsequent WGCNA analysis (Fig. 4A). Afterward, the network topology of 1 to 30 threshold weights was analyzed, and the soft threshold parameter β = 12 (R2>0.8) was selected to ensure a scale-free network (Fig. 4B). Subsequently, we screened 10 co-expression network modules, of which the gray module was composed of genes excluded from the other 9 modules (Fig. 4C). We analyzed the correlation of 10 modules with DKD and monocytes/macrophages, and then plotted the network heatmap. The result showed that the blue module was considered a key module, indicating a close correlation with DKD and macrophages M2 (Fig. 4D). 79 DEGs in the blue module, namely co-DEGs, were regarded as target genes for subsequent analysis (Fig. 4E).

### 3.4. Biofunctional and disease enrichment of genes in key modules

**Figure 5.**
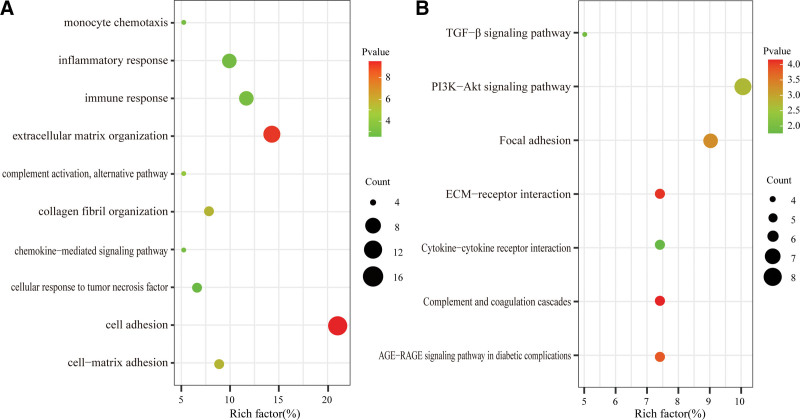
GO and KEGG enrichment analysis of co-DEGs. The color depth of the nodes refers to the *P* value. The size of the nodes refers to the number of genes (A) GO biological process (B) KEGG pathway. DEGs = differentially expressed genes, GO = gene ontology, KEGG = Kyoto encyclopedia of genes and genomes.

The biological function of co-DEGs in the blue module was studied by GO and KEGG enrichment analysis. The biological process of GO analysis showed that (Fig. 5A) target genes were mainly involved in cell adhesion, extractable matrix organization, collagen fiber organization, immune response, monocyte chemotaxis, and cellular response to tumor necrosis factor. In addition, KEGG analysis showed that (Fig. 5B) target genes were mainly enriched in immune response and fibrosis, such as completion and coagulation cascades, extracellular matrix (ECM)-receptor interaction, PI3K-Akt, and TGF-β signaling pathways.

### 3.5. PPI network construction and candidate genes screening

**Figure 6.**
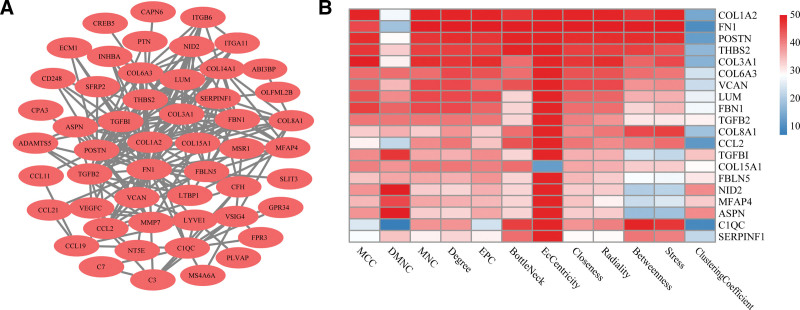
PPI network and identification of candidate genes. (A) PPI network of co-DEGs. (B) Heatmap of the CytoHubba analysis score. DEGs = differentially expressed genes, PPI = protein-protein interaction.

We constructed a PPI network to study the protein interaction of co-DEGs in the blue module. As shown in Figure 6A, the PPI network consisted of 52 nodes and 223 edges. The scores of all nodes for each algorithm were calculated by 12 topological analysis methods in CytoHubba and ranked from large to small, and then 1 to 50 points were allocated based on the ranking. According to the total score of each node, the top 20 nodes were selected as candidate genes, as shown in Figure 6B.

### 3.6. Hub-genes identification

**Figure 7.**
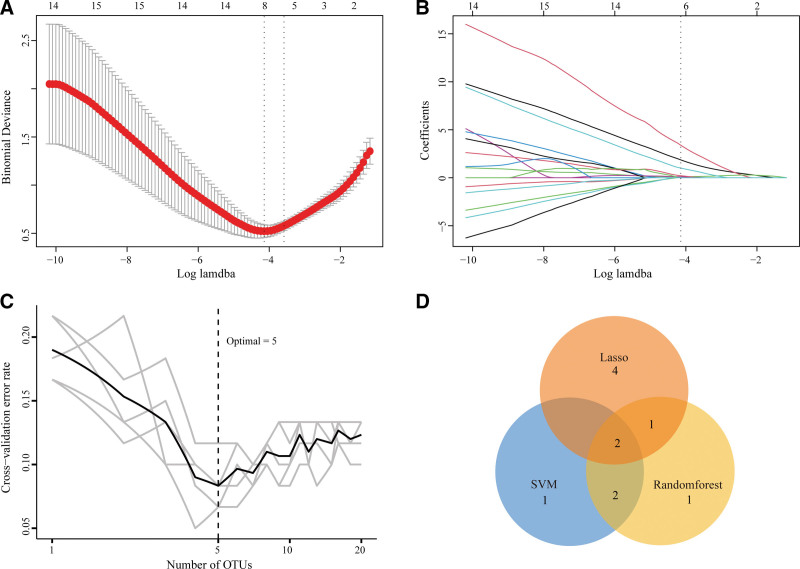
Identification of hub genes in DKD based on machine learning algorithms. (A) Optimal lambda of candidates selection in least absolute shrinkage and selection operator (LASSO) regression. (B) LASSO regression coefficient profiles of ten risk factors. (C) The optimum error rate of RF model based on 5 characteristic genes. (D) Venn diagram shows the overlapping genes in LASSO, support vector machines (SVM) and RF modules, namely hub genes. DKD = diabetic kidney disease, RF = randomforest.

We chose to apply machine learning algorithms to identify potential biomarkers of DKD from 20 candidates. First, the LASSO model was built based on DKD and control samples, with λ = 0.016. Thus, ASPN, C1QC, COL15A1, COL6A3, fibronectin 1 (FN1), LUM, POSTN, SERPINF1, transforming growth factor beta induced (TGFBI), and THBS2 were identified as potential genes for building the LASSO module (Fig. 7A and B). On the other hand, SVM-RFE analysis revealed that the SVM model based on 6 characteristic genes showed an optimum error rate. Therefore, FBLN5, TGFBI, NID2, LUM, FBN1, and FN1 were identified as potential genes. Meanwhile, the RF algorithm identified the top 5 genes from the 20 candidates, including FBN1, FN1, TGFBI, NID2, and COL6A3 (Fig. 7C). Finally, combining the results of the above 3 algorithms (Fig. 7D), FN1 and TGFBI were regarded as hub genes for DKD patients.

### 3.7. Hub-genes validation

**Figure 8.**
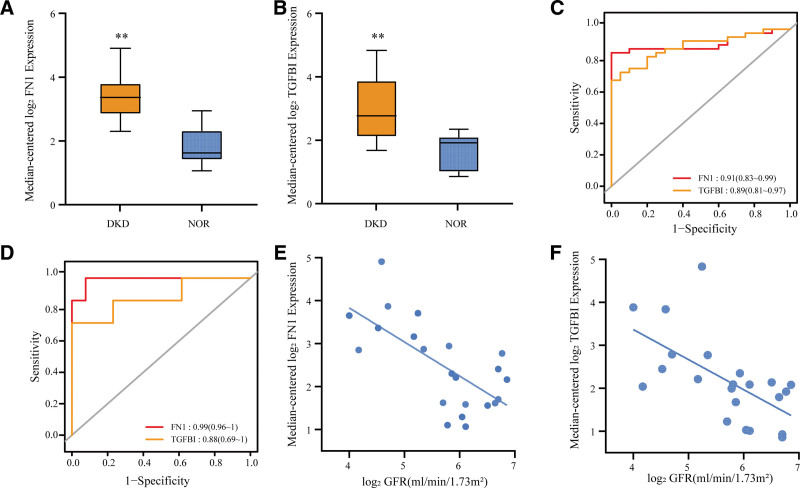
Validation of hub genes. (A and B) Gene expression levels of fibronectin 1 (FN1) and transforming growth factor beta induced (TGFBI) between DKD and healthy controls in GSE30528, respectively. ** *P* < .01. (C and D) Receiver operator characteristics (ROC) curves show hub genes in GSE96804 and validation in GSE30528, respectively. (E and F) Pearson correlation analysis of GFR and hub genes. FN1 and TGFBI are negatively related to GFR in DKD glomerulus samples (r = −0.68, *P* = 4.94 × 10^−4^, r = −0.744, *P* = 7.25 × 10^−5^). DKD = diabetic kidney disease.

To further evaluate the consistent changes of hub genes in DKD, we analyzed the validation dataset GSE30528. Compared with the controls, we found that FN1 and TGFBI mRNA levels in DKD patients were significantly higher (Fig. 8A and B). Furthermore, ROC analysis showed that the AUC values of FN1, TGFBI were >0.85 in both the GSE96804 and GSE30528 datasets (Fig. 8C and D), suggesting that they could be used as valid indicators for definitive diagnosis in DKD patients.

In order to verify the correlation between hub genes and renal function progression, the clinical data of GSE30528 were obtained from Nephroseq V5. Pearson correlation analysis showed that the expression levels of FN1 and TGFBI were negatively correlated with eGFR (r = −0.68, *P* = 4.94 × 10^−4^, r = −0.744, *P* = 7.25 × 10^−5^) (Fig. 8E and F).

**Figure 9.**
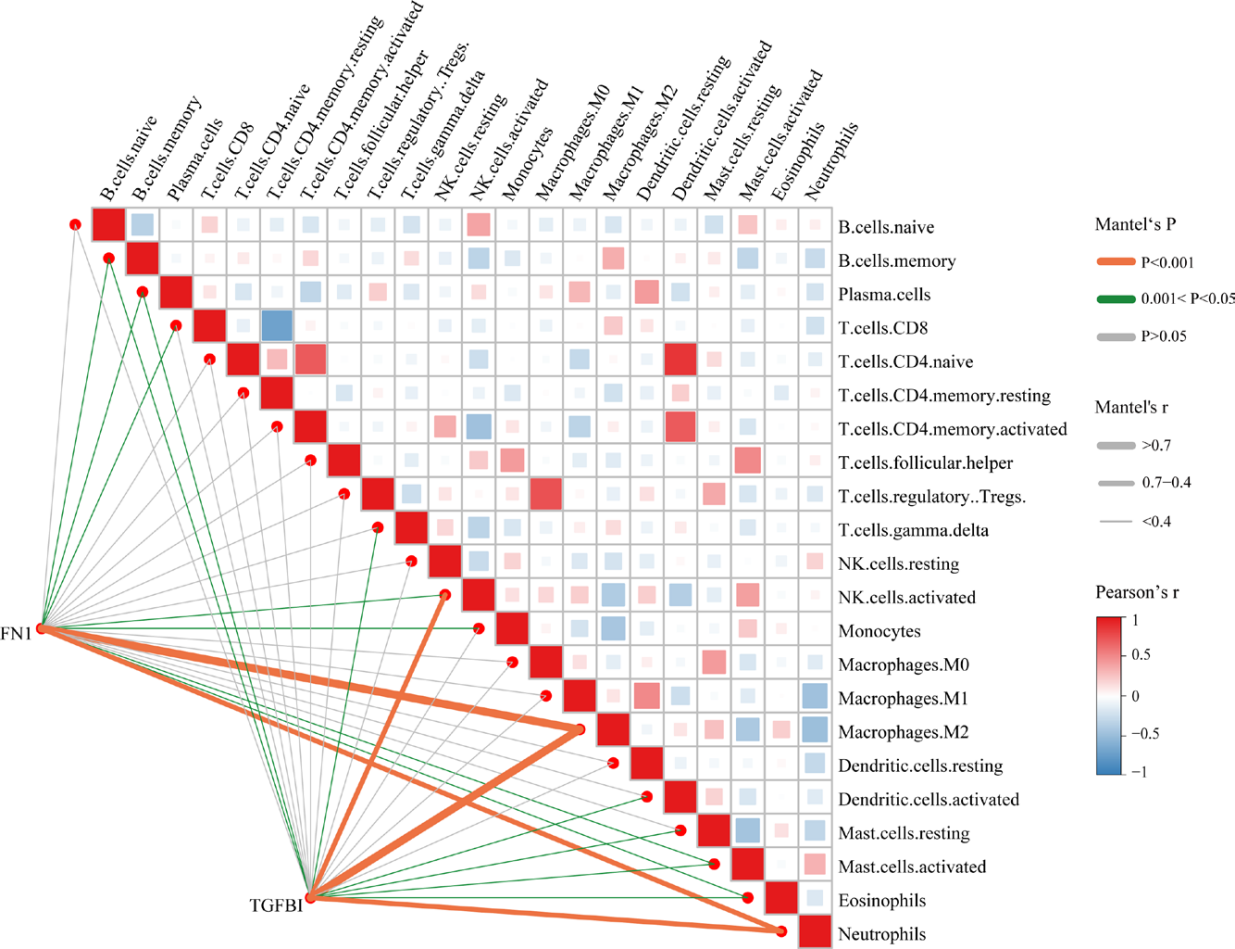
Correlation analysis between fibronectin 1 (FN1), transforming growth factor beta induced (TGFBI) and 22 kinds of immune cells. The color and the thickness of the line represents the *P* value and the correlation, respectively.

Correlation analysis showed that a strong positive correlation between FN1, TGFBI and Macrophages M2 (*R* > 0.7, *P* < .001) (Fig. 9).

## 4. Discussion

DKD is one of the microvascular complications of diabetes and is currently considered a chronic immune-inflammatory disease.^[31]^ With the depth of research, the role of macrophages in the pathogenesis of DKD has been recognized. In our study, CIBERSORTx was used to identify the infiltration of immune cells in the glomerular tissue of DKD. The results suggested that macrophage infiltration increased and accounted for a high proportion in the immune cells (M1 and M2), which was consistent with previous reports.

WGCNA is a comprehensive analysis technology based on a biological network, which divides potentially relevant genes into multiple modules and explores the correlation between key modules and clinical features to identify potential biomarkers or therapeutic targets.^[21]^ In this study, we found the blue module strongly associated with macrophage M2 infiltration in DKD samples by WGCNA, and identified a total of 79 target genes by crossing with DEGs. Interestingly, these 79 target genes were related to an extractable matrix organization, collagen fiber organization, immune response, monocyte chemotaxis and so on. Therefore, we speculate that these genes are involved in the immune response and fibrosis process induced by macrophages in the DKD state.

Numerous studies have shown that,^[32,33]^ macrophage accumulation could cause the progression of renal function and the deterioration of histopathological manifestations in DKD. After macrophage recruitment, it releases inflammatory mediators and stimulates the production of reactive oxygen species and proteases, resulting in tissue damage and fibrosis. Tumor necrosis factor-α in the kidney is mainly produced by macrophages.^[34]^ However, how macrophages of different phenotypes participate in the progression of DKD has not been fully defined.^[35]^ Traditionally, it is believed that macrophage M1 is involved in the initial stage of inflammation and is associated with tissue damage and pro-inflammatory function, whereas the M2 phenotype mediates repair and wound healing, mainly playing an anti-inflammatory and pro-angiogenic role. Both usually play opposite roles and coexist in DKD kidney tissue. Although some studies^[36,37]^ have reduced renal injury in DKD by inhibiting M1 activation and promoting M2 transformation, some studies have found that^[38]^ not all M2 therapies are beneficial and effective, and some M2 therapies can become pro-fibrotic. Notably, as revealed by Anders H.J. et al,^[39]^ the infiltration of macrophage M2 is positively correlated with the progression of renal fibrosis, and the possible mechanism is macrophage M2 to myofibroblast transition in the process, resulting in excessive deposition of ECM.^[40]^ However, it remains unclear how the M2 phenotype becomes pro-fibrotic and what the influence of the environment is. Therefore, macrophages are potential immune cell targets with research prospects, and regulation of the number of macrophages in the kidney and M1/M2 phenotypic transformation may play an important role in disease progression. At this stage, several studies have been carried out,^[41,42]^ with therapeutic strategies aimed at reducing macrophage infiltration or antibodies against downstream inflammatory mediators, thus reducing the progression of renal injury, but most remain at the stage of animal experiments.^[43]^ Therefore, actively exploring more specific biomarkers mediated macrophage induced immune injury in DKD remains an important issue that we urgently need to address.

Our study constructed a PPI network for 79 target genes, and subsequently used machine learning algorithms, namely LASSO logistic regression, SVM-RFE and RF algorithms to identify FN1, and TGFBI as hub genes in DKD. Next, we verified the diagnostic efficacy and prognostic risk of hub genes in the test cohort and clinical database, all showing statistical significance. Finally, the correlation results suggested that hub genes were strongly positively correlated with macrophage M2. Therefore, we hypothesize that FN1 and TGFBI are the diagnostic and prognostic markers of DKD, and may play a role in immune injury and fibrosis caused by macrophages.

FN1 is an ECM protein involved in various biological processes, such as cell adhesion, migration, differentiation and so on. It is well known that FN1, as a fibrogenic cytokine, plays an important role in the occurrence and progression of fibrosis in DKD.^[44,45]^ Recently, overexpression of FN1 has been found to promote disease progression in a variety of diseases, including heart failure^[46]^ and neoplastic diseases,^[47]^ and is associated with macrophage M2 polarization. It has been reported^[48]^ that FN1 promotes the secretion of macrophage M2 markers (IL-10) and reduces the production of M1 markers tumor necrosis factor-α in a concentration-dependent manner. In conjunction with our findings, it is speculated that in the development of DKD, FN1 may not only promote fibrosis, but also exert damaging effects by participating in macrophage polarization. It is a promising target for immunotherapy.

The results of our study showed that the expression of TGFBI was elevated in DKD patients, and the expression level was negatively correlated with eGFR, and significantly positively correlated with macrophage M2 cell infiltration. Combined with the research results of Robert J. Moritz et al,^[49]^ the hyperglycemic environment promotes macrophage infiltration, and macrophage derived TGF-β1 inducing TGFBI gene expression. The above-mentioned suggests that macrophage M2 infiltration may promote TGFBI gene expression in DKD patients. After the activation of the TGFBI gene, it encodes an ECM protein called BIGH3. The C-terminal portion of this protein is cleaved to derive an integrin-ligand peptide that induces apoptosis.^[50]^ The increase in the number of apoptotic cells further promotes macrophage infiltration, and macrophage uptake of apoptosis induces macrophage release of more TGF-β1. In addition, it can also stimulate the synthesis and secretion of BIGH3 protein by macrophages themselves, forming a potential feedback regulatory mechanism and promoting the progress of DKD.^[49,51]^ In addition, BIGH3 may also contribute to the progression of DKD by participating in the pro-fibrotic pathway driven by TGF-β1.^[52]^ In recent years, clinical studies have also reported that urinary BIGH3 levels are positively correlated with TGF-β and albumin excretion rate in patients with DKD,^[53]^ and are also elevated in diabetic patients with normal albuminuria.^[54]^ Urinary BIGH3 may become a promising early renal injury marker in DKD patients. In summary, we concluded that increased Macrophage M2 infiltration in DKD patients may promote the expression of TGFBI gene and the synthesis of BIGH3 protein, inducing renal cell apoptosis through the formation of a feedback regulatory mechanism, while promoting fibrosis, leading to the progression of DKD. It suggests that TGFBI may be involved in macrophage-induced immune injury and fibrosis.

However, there are some limitations to this study. First, the sample size from the GSE96804 dataset was limited and did not cover all patients with diabetic nephropathy. In addition, the microarray data were not classified according to the pathological stages of DKD, and the expression levels of certain genes may not be identical in different pathological stages. Finally, the specific molecular mechanisms and biological functions of these biomarkers remain to be validated by further experimental studies. Therefore, in the follow-up study, we will include more cases of DKD, including patients with different pathological stages, to verify the expression of hub genes in DKD patients, as well as the correlation between gene expression levels and clinical pathological stages. In addition, we will demonstrate the expression levels of FN1 and TGFBI at the cellular and animal experimental levels, further investigate the correlation between genes in DKD and macrophage expression, and further validate the actual regulatory function of genes and their impact on functional phenotype through subsequent experiments. This will provide new directions and therapeutic targets for the future treatment of DKD from the perspective of macrophages and immune inflammation.

## 5. Conclusion

In this study, we applied immune infiltration analysis, WGCNA combined with machine learning algorithms to reveal the biological function of macrophages in the progression of DKD, that is, they participate in the immune response and fibrosis process. We identified FN1 and TGFBI as promising biomarkers, and validated their good diagnostic effect and prognostic value. It will help to provide potential new therapeutic targets for DKD from the perspective of macrophages and immune inflammation in the future.

## Acknowledgments

We thank the researchers and patients who contributed data to the GEO database.

## Author contributions

**Conceptualization:** Fulin Dou.

**Formal analysis:** Fulin Dou.

**Data curation:** Qingzhen Liu, Shasha Lv.

**Writing – original draft:** Qiaoying Xu, Xueling Wang.

**Writing – review & editing:** Shanshan Liu, Gang Liu.
